# Supplementary material for: A Likelihood Approach for Real-Time Calibration of Stochastic Compartmental Epidemic Models
Source: PLoS Comput Biol. 2017 Jan 17;13(1):e1005257. doi: 10.1371/journal.pcbi.1005257 (PMC5240920; doi:10.1371/journal.pcbi.1005257)
Supplement: S1 File — (TAR.GZ) [file pcbi.1005257.s014.tar.gz › HSPH_Online-SI-Revision/PF08/n100-mild/n100-mild_PF2_table8.pdf]

1	newly 3 weeks spec	114.	2872.98	{2795., 2951.}	2420.16	1.40135	newly 3 weeks cumul	602.	10363.	{10287., 10450.}	1621.42	1.23588	newly 5 weeks spec	30.	1971.56	{1914., 2029.}	6471.87	1.81757	newly 5 weeks cumul	708.	14721.5	{14619., 14833.}	1979.31	1.31791	newly 44 weeks cumul	814.	22938.4	{22722., 23113.}	2717.99	1.44993
2	newly 3 weeks spec	204.	2057.78	{1993., 2132.}	908.716	1.00363	newly 3 weeks cumul	827.	7571.39	{7453., 7688.}	815.525	0.961638	newly 5 weeks spec	99.	1347.7	{1295., 1398.}	1261.31	1.13373	newly 5 weeks cumul	1036.	10597.8	{10440., 10777.}	922.958	1.00983	newly 43 weeks cumul	1187.	15753.8	{15497., 16024.}	1227.19	1.12289
3	newly 3 weeks spec	108.	2802.87	{2720., 2872.}	2495.25	1.4141	newly 3 weeks cumul	712.	10035.3	{9949., 10119.}	1309.45	1.14904	newly 5 weeks spec	44.	1914.61	{1859., 1964.}	4251.39	1.63852	newly 5 weeks cumul	820.	14273.5	{14138., 14409.}	1640.66	1.2407	newly 46 weeks cumul	845.	22193.8	{21906., 22467.}	2526.49	1.41935
4	newly 3 weeks spec	19.	1199.45	{1160., 1239.}	6212.89	1.80007	newly 3 weeks cumul	149.	4822.89	{4753., 4882.}	3136.84	1.5101	newly 5 weeks spec	0.1	691.75	{660., 717.}	691650.	3.83966	newly 5 weeks cumul	155.	6431.	{6350., 6503.}	4049.03	1.61793	newly 50 weeks cumul	155.	8598.34	{8466., 8689.}	5447.32	1.74406
5	newly 3 weeks spec	147.	2024.97	{1962., 2077.}	1277.53	1.13899	newly 3 weeks cumul	785.	7607.1	{7533., 7687.}	869.057	0.986334	newly 5 weeks spec	78.	1301.54	{1256., 1346.}	1568.64	1.2222	newly 5 weeks cumul	977.	10535.6	{10439., 10635.}	978.357	1.03275	newly 46 weeks cumul	1144.	15353.3	{15114., 15560.}	1242.07	1.12775
6	newly 3 weeks spec	116.	2050.34	{1987., 2117.}	1667.53	1.24726	newly 3 weeks cumul	649.	7527.92	{7443., 7601.}	1059.93	1.06442	newly 5 weeks spec	30.	1364.17	{1315., 1414.}	4447.23	1.65756	newly 5 weeks cumul	747.	10571.3	{10459., 10667.}	1315.16	1.15079	newly 44 weeks cumul	826.	16125.9	{15918., 16345.}	1852.29	1.29052
7	newly 3 weeks spec	14.	1785.31	{1730., 1839.}	12652.2	2.10547	newly 3 weeks cumul	126.	7146.41	{7073., 7214.}	5571.75	1.7537	newly 5 weeks spec	0.1	1040.09	{982., 1086.}	1.03999×10^6	4.01682	newly 5 weeks cumul	128.	9551.41	{9476., 9615.}	7362.04	1.87285	newly 49 weeks cumul	128.	12789.9	{12698., 12867.}	9892.07	1.99965
8	newly 3 weeks spec	39.	2344.27	{2285., 2401.}	5910.95	1.77886	newly 3 weeks cumul	302.	8600.69	{8527., 8678.}	2747.91	1.45451	newly 5 weeks spec	15.	1564.45	{1511., 1621.}	10329.7	2.0181	newly 5 weeks cumul	333.	12092.5	{11996., 12208.}	3531.37	1.56006	newly 43 weeks cumul	361.	18439.1	{18199., 18718.}	5007.77	1.70821
9	newly 3 weeks spec	85.	1426.33	{1382., 1466.}	1578.04	1.22466	newly 3 weeks cumul	398.	5715.09	{5662., 5775.}	1335.95	1.15712	newly 5 weeks spec	41.	822.07	{786., 862.}	1905.05	1.30183	newly 5 weeks cumul	509.	7619.09	{7540., 7708.}	1396.87	1.17517	newly 48 weeks cumul	521.	10157.2	{10043., 10286.}	1849.55	1.28992
10	newly 3 weeks spec	178.	1956.52	{1898., 2022.}	999.169	1.04093	newly 3 weeks cumul	763.	6917.6	{6835., 6986.}	806.632	0.957413	newly 5 weeks spec	52.	1397.85	{1335., 1451.}	2588.17	1.42922	newly 5 weeks cumul	913.	9976.71	{9875., 10082.}	992.739	1.0385	newly 42 weeks cumul	1004.	16692.4	{16426., 16936.}	1562.59	1.22076
11	newly 3 weeks spec	68.	2046.77	{1974., 2102.}	2909.96	1.47844	newly 3 weeks cumul	409.	7836.69	{7748., 7932.}	1816.06	1.28239	newly 5 weeks spec	9.	1262.47	{1215., 1312.}	13927.4	2.14675	newly 5 weeks cumul	430.	10710.4	{10612., 10814.}	2390.79	1.39632	newly 46 weeks cumul	447.	15105.	{14900., 15307.}	3279.19	1.52879
12	newly 3 weeks spec	387.	1901.51	{1840., 1961.}	391.346	0.691262	newly 3 weeks cumul	1436.	7011.21	{6930., 7090.}	388.246	0.688622	newly 5 weeks spec	224.	1258.88	{1208., 1303.}	462.	0.749524	newly 5 weeks cumul	1967.	9822.78	{9705., 9921.}	399.379	0.698415	newly 42 weeks cumul	2777.	14892.8	{14631., 15146.}	436.292	0.729367
13	newly 3 weeks spec	44.	1418.5	{1359., 1469.}	3123.86	1.50816	newly 3 weeks cumul	425.	5776.45	{5677., 5876.}	1259.16	1.13323	newly 5 weeks spec	11.	803.83	{760., 850.}	7207.55	1.86336	newly 5 weeks cumul	440.	7651.34	{7496., 7777.}	1638.94	1.24025	newly 47 weeks cumul	442.	9938.46	{9686., 10164.}	2148.52	1.35182
14	newly 3 weeks spec	73.	1388.06	{1343., 1427.}	1801.45	1.27897	newly 3 weeks cumul	262.	5476.98	{5410., 5536.}	1990.45	1.32022	newly 5 weeks spec	18.	822.73	{780., 863.}	4470.72	1.65965	newly 5 weeks cumul	346.	7373.49	{7308., 7429.}	2031.07	1.32859	newly 49 weeks cumul	354.	10097.2	{10021., 10176.}	2752.31	1.45519
15	newly 3 weeks spec	78.	1783.58	{1733., 1829.}	2186.64	1.35908	newly 3 weeks cumul	442.	6988.87	{6920., 7062.}	1481.19	1.19897	newly 5 weeks spec	22.	1067.67	{1024., 1116.}	4753.05	1.6858	newly 5 weeks cumul	504.	9443.54	{9379., 9521.}	1773.72	1.2727	newly 46 weeks cumul	595.	12957.5	{12803., 13104.}	2077.74	1.33799
16	newly 3 weeks spec	250.	2762.67	{2680., 2838.}	1005.07	1.04326	newly 3 weeks cumul	1232.	9435.22	{9340., 9532.}	665.846	0.884127	newly 5 weeks spec	117.	2071.68	{2004., 2138.}	1670.67	1.24801	newly 5 weeks cumul	1525.	13914.2	{13767., 14064.}	812.406	0.960173	newly 42 weeks cumul	1724.	24453.1	{24096., 24821.}	1318.39	1.15177
17	newly 3 weeks spec	47.	1530.31	{1467., 1605.}	3155.98	1.51243	newly 3 weeks cumul	382.	6291.03	{6142., 6418.}	1546.87	1.2166	newly 5 weeks spec	36.	846.81	{794., 897.}	2252.25	1.37099	newly 5 weeks cumul	445.	8282.73	{8071., 8468.}	1761.29	1.26973	newly 48 weeks cumul	515.	10618.4	{10252., 10951.}	1961.83	1.3141
18	newly 3 weeks spec	0.1	1692.5	{1640., 1739.}	1.6924×10^6	4.22841	newly 3 weeks cumul	28.	6839.54	{6775., 6901.}	24326.9	2.38786	newly 5 weeks spec	0.1	974.86	{935., 1015.}	974760.	3.98872	newly 5 weeks cumul	28.	9100.78	{9039., 9151.}	32402.8	2.51192	newly 52 weeks cumul	28.	12075.1	{12015., 12134.}	43025.3	2.63473
19	newly 3 weeks spec	126.	2646.78	{2586., 2716.}	2000.62	1.32227	newly 3 weeks cumul	683.	9465.79	{9392., 9559.}	1285.91	1.14173	newly 5 weeks spec	78.	1849.14	{1784., 1912.}	2270.69	1.37475	newly 5 weeks cumul	871.	13534.3	{13431., 13641.}	1453.89	1.19141	newly 43 weeks cumul	985.	21544.8	{21267., 21823.}	2087.29	1.33988
20	newly 3 weeks spec	0.1	1817.88	{1767., 1868.}	1.81778×10^6	4.25946	newly 3 weeks cumul	49.	7398.23	{7324., 7461.}	14998.4	2.17892	newly 5 weeks spec	0.1	1023.72	{974., 1073.}	1.02362×10^6	4.00991	newly 5 weeks cumul	49.	9794.26	{9734., 9851.}	19888.3	2.30077	newly 52 weeks cumul	49.	12825.2	{12754., 12909.}	26073.8	2.41786
21	newly 3 weeks spec	25.	1476.97	{1437., 1520.}	5807.88	1.77132	newly 3 weeks cumul	110.	6260.32	{6204., 6310.}	5591.2	1.75519	newly 5 weeks spec	10.	764.78	{733., 798.}	7547.8	1.88327	newly 5 weeks cumul	146.	8084.37	{8008., 8164.}	5437.24	1.74328	newly 52 weeks cumul	154.	10001.1	{9862., 10140.}	6394.25	1.81251
22	newly 3 weeks spec	390.	2086.04	{2028., 2149.}	434.882	0.728151	newly 3 weeks cumul	1699.	7390.18	{7294., 7477.}	334.972	0.638439	newly 5 weeks spec	240.	1479.38	{1423., 1535.}	516.408	0.789662	newly 5 weeks cumul	2286.	10631.8	{10514., 10744.}	365.082	0.667513	newly 42 weeks cumul	2969.	17543.4	{17246., 17875.}	490.885	0.771452
23	newly 3 weeks spec	51.	2036.14	{1981., 2092.}	3892.43	1.60113	newly 3 weeks cumul	271.	7993.75	{7893., 8075.}	2849.72	1.46977	newly 5 weeks spec	0.1	1197.08	{1146., 1249.}	1.19698×10^6	4.07783	newly 5 weeks cumul	288.	10756.3	{10649., 10850.}	3634.84	1.57226	newly 47 weeks cumul	288.	14509.4	{14342., 14692.}	4938.	1.70224
24	newly 3 weeks spec	1.	2008.23	{1947., 2068.}	200723.	3.30269	newly 3 weeks cumul	56.	7372.01	{7289., 7446.}	13064.3	2.11938	newly 5 weeks spec	0.1	1356.57	{1303., 1409.}	1.35647×10^6	4.13222	newly 5 weeks cumul	56.	10389.5	{10287., 10479.}	18452.6	2.26839	newly 51 weeks cumul	56.	16188.6	{16006., 16369.}	28808.2	2.461
25	newly 3 weeks spec	333.	2600.	{2519., 2682.}	680.781	0.892393	newly 3 weeks cumul	1455.	9104.18	{8940., 9235.}	525.717	0.79635	newly 5 weeks spec	128.	1871.24	{1791., 1931.}	1361.91	1.16474	newly 5 weeks cumul	1803.	13179.7	{12909., 13334.}	630.989	0.863882	newly 42 weeks cumul	2085.	22205.3	{21454., 22625.}	965.001	1.02727
26	newly 3 weeks spec	134.	2038.61	{1982., 2097.}	1421.35	1.18213	newly 3 weeks cumul	702.	7584.54	{7497., 7678.}	980.419	1.03357	newly 5 weeks spec	48.	1327.48	{1277., 1377.}	2665.58	1.44162	newly 5 weeks cumul	832.	10565.	{10472., 10683.}	1169.83	1.10373	newly 44 weeks cumul	1092.	15739.3	{15526., 15963.}	1341.33	1.15874
27	newly 3 weeks spec	208.	2584.93	{2513., 2666.}	1142.75	1.09427	newly 3 weeks cumul	1021.	9080.2	{8925., 9190.}	789.344	0.949042	newly 5 weeks spec	93.	1852.05	{1770., 1927.}	1891.45	1.29897	newly 5 weeks cumul	1280.	13121.5	{12831., 13292.}	925.113	1.01074	newly 42 weeks cumul	1520.	21940.3	{21182., 22297.}	1343.44	1.15932
28	newly 3 weeks spec	419.	2519.08	{2448., 2598.}	501.212	0.77892	newly 3 weeks cumul	1620.	8787.51	{8685., 8869.}	442.439	0.734334	newly 5 weeks spec	296.	1804.62	{1741., 1864.}	509.669	0.784945	newly 5 weeks cumul	2248.	12722.9	{12592., 12855.}	465.963	0.752773	newly 43 weeks cumul	3114.	21290.3	{20940., 21647.}	583.697	0.834824
29	newly 3 weeks spec	193.	2814.39	{2737., 2875.}	1358.23	1.16375	newly 3 weeks cumul	755.	10070.3	{9991., 10146.}	1233.81	1.12509	newly 5 weeks spec	61.	1942.76	{1875., 2013.}	3084.85	1.50293	newly 5 weeks cumul	915.	14361.3	{14230., 14482.}	1469.54	1.19576	newly 45 weeks cumul	1584.	22495.8	{22175., 22804.}	1320.19	1.15232
30	newly 3 weeks spec	34.	1148.68	{1106., 1196.}	3278.47	1.52851	newly 3 weeks cumul	205.	4866.37	{4799., 4929.}	2273.84	1.37543	newly 5 weeks spec	6.	602.15	{564., 632.}	9935.83	2.00113	newly 5 weeks cumul	220.	6299.02	{6226., 6370.}	2763.19	1.45683	newly 47 weeks cumul	225.	7859.49	{7757., 7968.}	3393.11	1.54319
31	newly 3 weeks spec	208.	2658.24	{2574., 2728.}	1178.	1.10643	newly 3 weeks cumul	1042.	9670.27	{9598., 9751.}	828.049	0.967561	newly 5 weeks spec	80.	1775.07	{1710., 1835.}	2118.84	1.34597	newly 5 weeks cumul	1231.	13621.7	{13489., 13726.}	1006.55	1.04396	newly 44 weeks cumul	1394.	20531.	{20240., 20771.}	1372.81	1.16812
32	newly 3 weeks spec	553.	1913.64	{1855., 1978.}	246.047	0.539017	newly 3 weeks cumul	1991.	6935.07	{6839., 7059.}	248.321	0.541948	newly 5 weeks spec	288.	1314.01	{1247., 1379.}	356.253	0.658923	newly 5 weeks cumul	2687.	9843.96	{9700., 10021.}	266.355	0.563869	newly 42 weeks cumul	3562.	15547.4	{15226., 15966.}	336.48	0.6399
33	newly 3 weeks spec	0.1	1967.06	{1919., 2014.}	1.96696×10^6	4.29373	newly 3 weeks cumul	6.	7967.9	{7904., 8041.}	132698.	3.12318	newly 5 weeks spec	0.1	1115.86	{1080., 1153.}	1.11576×10^6	4.04746	newly 5 weeks cumul	6.	10566.4	{10506., 10626.}	176007.	3.24577	newly 52 weeks cumul	6.	13884.7	{13824., 13946.}	231312.	3.36438
34	newly 3 weeks spec	24.	1325.2	{1282., 1367.}	5421.67	1.74194	newly 3 weeks cumul	66.	5332.89	{5271., 5390.}	7980.14	1.9074	newly 5 weeks spec	13.	770.66	{733., 807.}	5828.15	1.77263	newly 5 weeks cumul	105.	7115.78	{7061., 7168.}	6676.93	1.83103	newly 48 weeks cumul	110.	9529.14	{9461., 9598.}	8562.85	1.93765
35	newly 3 weeks spec	0.1	2267.57	{2205., 2331.}	2.26747×10^6	4.35546	newly 3 weeks cumul	0.1	9118.32	{9043., 9179.}	9.11822×10^6	4.95991	newly 5 weeks spec	0.1	1290.79	{1238., 1338.}	1.29069×10^6	4.11067	newly 5 weeks cumul	0.1	12119.	{12039., 12190.}	1.21189×10^7	5.08346	newly 52 weeks cumul	0.1	16020.1	{15942., 16111.}	1.602×10^7	5.20466
36	newly 3 weeks spec	107.	2356.05	{2298., 2419.}	2101.92	1.34272	newly 3 weeks cumul	314.	9289.84	{9213., 9372.}	2858.55	1.47107	newly 5 weeks spec	47.	1389.25	{1341., 1438.}	2855.85	1.4705	newly 5 weeks cumul	444.	12493.7	{12408., 12557.}	2713.91	1.4493	newly 52 weeks cumul	542.	16935.7	{16861., 17016.}	3024.66	1.4948
37	newly 3 weeks spec	66.	1810.65	{1763., 1862.}	2643.41	1.43818	newly 3 weeks cumul	452.	6930.51	{6850., 7016.}	1433.3	1.18561	newly 5 weeks spec	38.	1140.87	{1085., 1190.}	2902.29	1.4772	newly 5 weeks cumul	556.	9515.51	{9412., 9606.}	1611.42	1.23334	newly 45 weeks cumul	581.	13590.4	{13392., 13756.}	2239.13	1.36903
38	newly 3 weeks spec	0.1	1750.37	{1703., 1795.}	1.75027×10^6	4.24304	newly 3 weeks cumul	6.	7107.52	{7037., 7171.}	118359.	3.07355	newly 5 weeks spec	0.1	991.61	{955., 1023.}	991510.	3.99618	newly 5 weeks cumul	6.	9423.82	{9359., 9481.}	156964.	3.19607	newly 52 weeks cumul	6.	12383.8	{12312., 12453.}	206297.	3.3147
39	newly 3 weeks spec	62.	1421.02	{1378., 1463.}	2191.97	1.36006	newly 3 weeks cumul	248.	5769.78	{5714., 5826.}	2226.52	1.3667	newly 5 weeks spec	8.	807.96	{771., 843.}	9999.5	2.00404	newly 5 weeks cumul	278.	7649.53	{7578., 7709.}	2651.63	1.43958	newly 49 weeks cumul	290.	10034.7	{9943., 10120.}	3360.26	1.5391
40	newly 3 weeks spec	1.	1336.22	{1298., 1371.}	133522.	3.12575	newly 3 weeks cumul	22.	5483.27	{5414., 5546.}	24824.	2.3966	newly 5 weeks spec	0.1	749.9	{712., 785.}	749800.	3.87472	newly 5 weeks cumul	22.	7237.07	{7180., 7290.}	32795.8	2.51713	newly 51 weeks cumul	22.	9469.78	{9408., 9528.}	42944.5	2.63391
41	newly 3 weeks spec	9.	1628.23	{1572., 1677.}	17991.4	2.25734	newly 3 weeks cumul	150.	6576.25	{6504., 6641.}	4284.17	1.64187	newly 5 weeks spec	0.1	942.08	{900., 974.}	941980.	3.97391	newly 5 weeks cumul	158.	8754.94	{8685., 8823.}	5441.1	1.74359	newly 52 weeks cumul	158.	11618.5	{11547., 11689.}	7253.47	1.86649
42	newly 3 weeks spec	40.	1451.52	{1408., 1490.}	3528.8	1.55966	newly 3 weeks cumul	178.	5930.3	{5859., 5988.}	3231.63	1.52264	newly 5 weeks spec	2.	816.18	{774., 851.}	40709.	2.61047	newly 5 weeks cumul	187.	7837.38	{7778., 7901.}	4091.11	1.62232	newly 49 weeks cumul	187.	10221.3	{10135., 10302.}	5365.95	1.73766
43	newly 3 weeks spec	34.	1631.97	{1569., 1681.}	4699.91	1.6811	newly 3 weeks cumul	177.	6579.29	{6516., 6642.}	3617.11	1.57019	newly 5 weeks spec	7.	942.41	{902., 976.}	13363.	2.12891	newly 5 weeks cumul	204.	8758.92	{8685., 8826.}	4193.59	1.63281	newly 52 weeks cumul	209.	11629.3	{11544., 11704.}	5464.27	1.7454
44	newly 3 weeks spec	5.	1468.22	{1427., 1510.}	29264.4	2.4677	newly 3 weeks cumul	94.	5997.43	{5938., 6065.}	6280.24	1.80482	newly 5 weeks spec	0.1	826.42	{786., 863.}	826320.	3.91693	newly 5 weeks cumul	94.	7924.96	{7865., 7980.}	8330.81	1.92586	newly 51 weeks cumul	94.	10359.4	{10296., 10423.}	10920.6	2.0422
45	newly 3 weeks spec	93.	1761.17	{1701., 1823.}	1793.73	1.27717	newly 3 weeks cumul	558.	6807.7	{6712., 6892.}	1120.02	1.08635	newly 5 weeks spec	65.	1068.32	{1016., 1111.}	1543.57	1.21549	newly 5 weeks cumul	698.	9253.99	{9127., 9352.}	1225.79	1.12246	newly 47 weeks cumul	726.	12788.7	{12606., 13012.}	1661.53	1.24585
46	newly 3 weeks spec	37.	1655.04	{1599., 1705.}	4373.08	1.65047	newly 3 weeks cumul	384.	6767.91	{6709., 6835.}	1662.48	1.24611	newly 5 weeks spec	4.	910.95	{871., 947.}	22673.8	2.35718	newly 5 weeks cumul	404.	8920.56	{8840., 8995.}	2108.06	1.344	newly 48 weeks cumul	409.	11500.4	{11353., 11657.}	2711.84	1.44897
47	newly 3 weeks spec	33.	1414.92	{1361., 1467.}	4187.64	1.63204	newly 3 weeks cumul	146.	5679.8	{5619., 5735.}	3790.27	1.58996	newly 5 weeks spec	17.	815.55	{776., 856.}	4697.35	1.6807	newly 5 weeks cumul	191.	7563.23	{7503., 7619.}	3859.81	1.59767	newly 48 weeks cumul	201.	10089.4	{9997., 10157.}	4919.58	1.70066
48	newly 3 weeks spec	101.	2862.6	{2789., 2939.}	2734.26	1.45235	newly 3 weeks cumul	455.	9990.01	{9850., 10100.}	2095.61	1.34153	newly 5 weeks spec	16.	2065.38	{1993., 2130.}	12808.6	2.11073	newly 5 weeks cumul	527.	14492.7	{14273., 14657.}	2650.03	1.43931	newly 43 weeks cumul	536.	24323.	{23509., 24786.}	4437.88	1.65678
49	newly 3 weeks spec	21.	1571.33	{1519., 1624.}	7382.52	1.87388	newly 3 weeks cumul	128.	6387.56	{6321., 6460.}	4890.28	1.69811	newly 5 weeks spec	0.1	879.24	{832., 918.}	879140.	3.9438	newly 5 weeks cumul	131.	8447.96	{8333., 8541.}	6348.82	1.80946	newly 49 weeks cumul	131.	10970.3	{10798., 11136.}	8274.26	1.92292
50	newly 3 weeks spec	8.	1280.91	{1240., 1317.}	15911.4	2.2043	newly 3 weeks cumul	96.	5114.36	{5060., 5168.}	5227.46	1.7265	newly 5 weeks spec	0.1	746.49	{710., 781.}	746390.	3.87268	newly 5 weeks cumul	102.	6836.81	{6778., 6884.}	6602.75	1.82624	newly 50 weeks cumul	102.	9202.14	{9135., 9265.}	8921.71	1.95528
